# Supplementary material for: HIV vaccine candidate ΔV1gp120 formulated in ALFQA adjuvant augments mucosal immunity in female macaques
Source: Nat Commun. 2025 Sep 29;16:8571. doi: 10.1038/s41467-025-63610-z (PMC12480912; doi:10.1038/s41467-025-63610-z)
Supplement: Supplementary file 1 — Supplementary information [file 41467_2025_63610_MOESM1_ESM.pdf]

Supplementary Information

SUPPLEMENTARY FIGURES

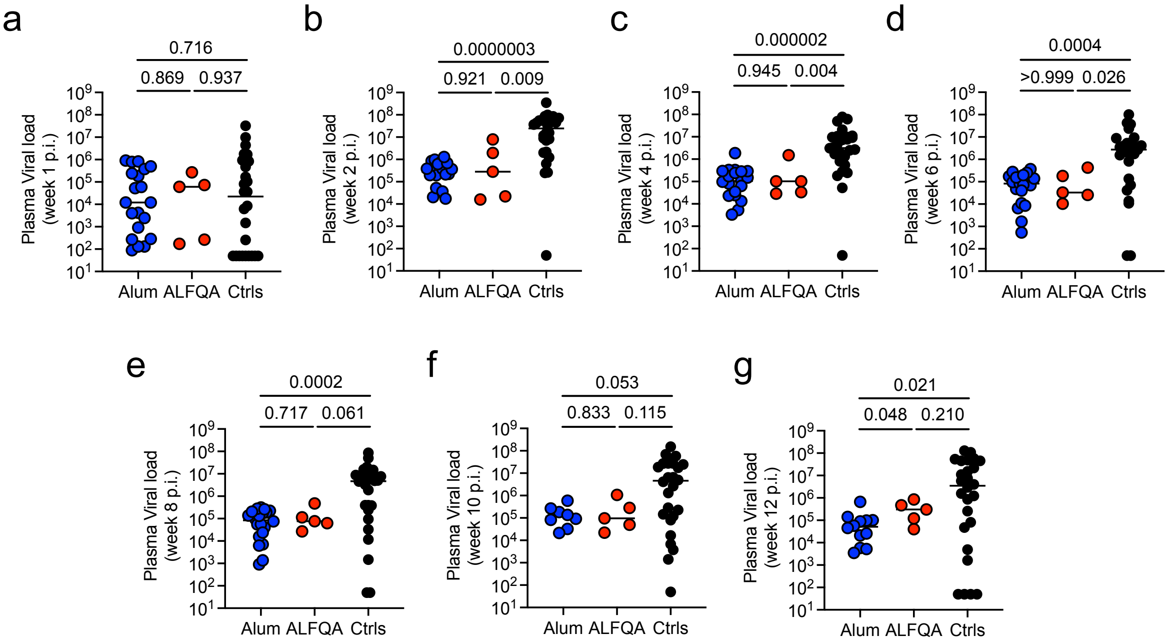

Supplementary Figure 1

Supplementary Figure 1. Viral load. (**a-g**) Log_{10} Simian Immunodeficiency Virus (SIV) RNA levels in plasma at (**a**) 1, (**b**) 2, (**c**) 4, (**d**) 6, (**e**) 8, (**f**) 10 and (**g**) 12 weeks following SIV_{mac251} infection in Alum (n=20 in **a, b, d** and **e**; n=19, **c**; n=8, **f**; n=12, **g**), ALFQA (n=5, **a-g**), and control (n=26, **a-g**) animals. Comparisons: (**a-g**) two-tailed Mann-Whitney U test with median. P-values are unadjusted for multiple comparisons. In panels a-g, SIV-immunized Alum and ALFQA animals are depicted as black circles filled in blue and red, respectively, whereas control animals are depicted as black circles. Source data are provided as a Source Data file.

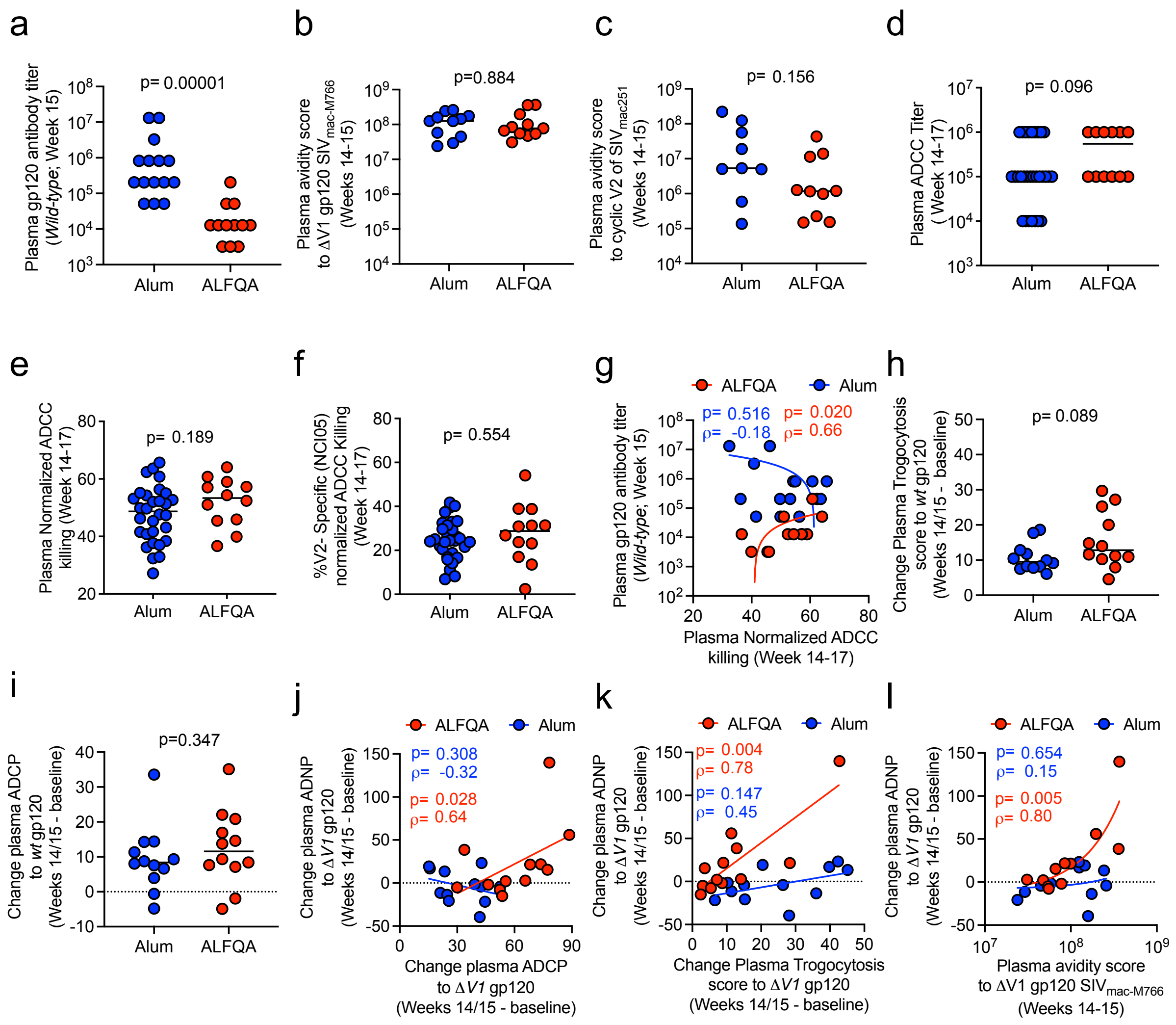

Supplementary Figure 2

Supplementary Figure 2. Antibody titers and Fc receptor-dependent function of antibodies. (**a**) Log_{10} Serum IgG antibody titers to whole SIV_{m766} wild type gp120 protein in Alum (n=15) and ALFQA (n=12) animals at week 15. (**b, c**) Plasma Log_{10} avidity score of antibodies targeting (**b**) the whole SIV_{m766} ΔV1gp120 protein and (**c**) the cyclic V2 region of SIV_{mac251} in Alum (n=11 and 9, respectively) and ALFQA (n=11 and 10, respectively) animals at weeks 14-15. (**d, e**) Plasma (**d**) Log_{10} titers of antibodies mediating antibody-dependent cellular cytotoxicity (ADCC) and (**e**) normalized ADCC killing targeting gp120 coated cells in Alum (n=30) and ALFQA

(n=12) at weeks 14-17. (**f**) V2-specific ADCC against gp120 coated cells in plasma of Alum (n=30) and ALFQA (n=12) animals at week 14-17. V2-specific ADCC was assessed using $F(ab')_2$ of NCI05 antibody targeting V2. (**g**) Correlation between Log_{10} serum IgG antibody titers to whole SIV_{m766} wild type gp120 protein at week 15 and plasma normalized gp120 ADCC killing at week 14/17 of Alum (n=15) and ALFQA (n=12) animals. (**h, i**) Vaccine-induced change (week 14/15 – baseline) of (**h**) trogocytosis score and (**i**) antibody-dependent cellular phagocytosis (ADCP) against SIV_{m766} wild type gp120 protein measured in plasma of Alum (n=12) and ALFQA (n=12) animals. (**j, k**) Correlations between vaccine-induced changes (week 14/15 – baseline) antibody-dependent neutrophil phagocytosis (ADNP) and (**j**) ADCP or (**k**) trogocytosis score against SIV_{m766} ΔV1gp120 protein measured in plasma in Alum (n=12) and ALFQA (n=12) animals. (**l**) Correlation between vaccine-induced changes (week 14/15 – baseline) ADNP against SIV_{m766} ΔV1gp120 protein and Log_{10} avidity score of antibodies targeting the whole SIV_{m766} ΔV1gp120 protein at week 14/15 measured in plasma in Alum (n=11) and ALFQA (n=11) animals.
Comparisons: (**a-f, h, i**) two-tailed Mann-Whitney U test with median; Correlations: (**g, j-l**) two-tailed Spearman correlation with simple linear regression. Alum animals and correlations are depicted as black circles filled in blue and blue lines, ALFQA animals and correlations are depicted as black circles filled in in red and red lines. In panels a-f and h-i, SIV-immunized Alum and ALFQA animals are depicted as black circles filled in blue and red, respectively. Source data are provided as a Source Data file.

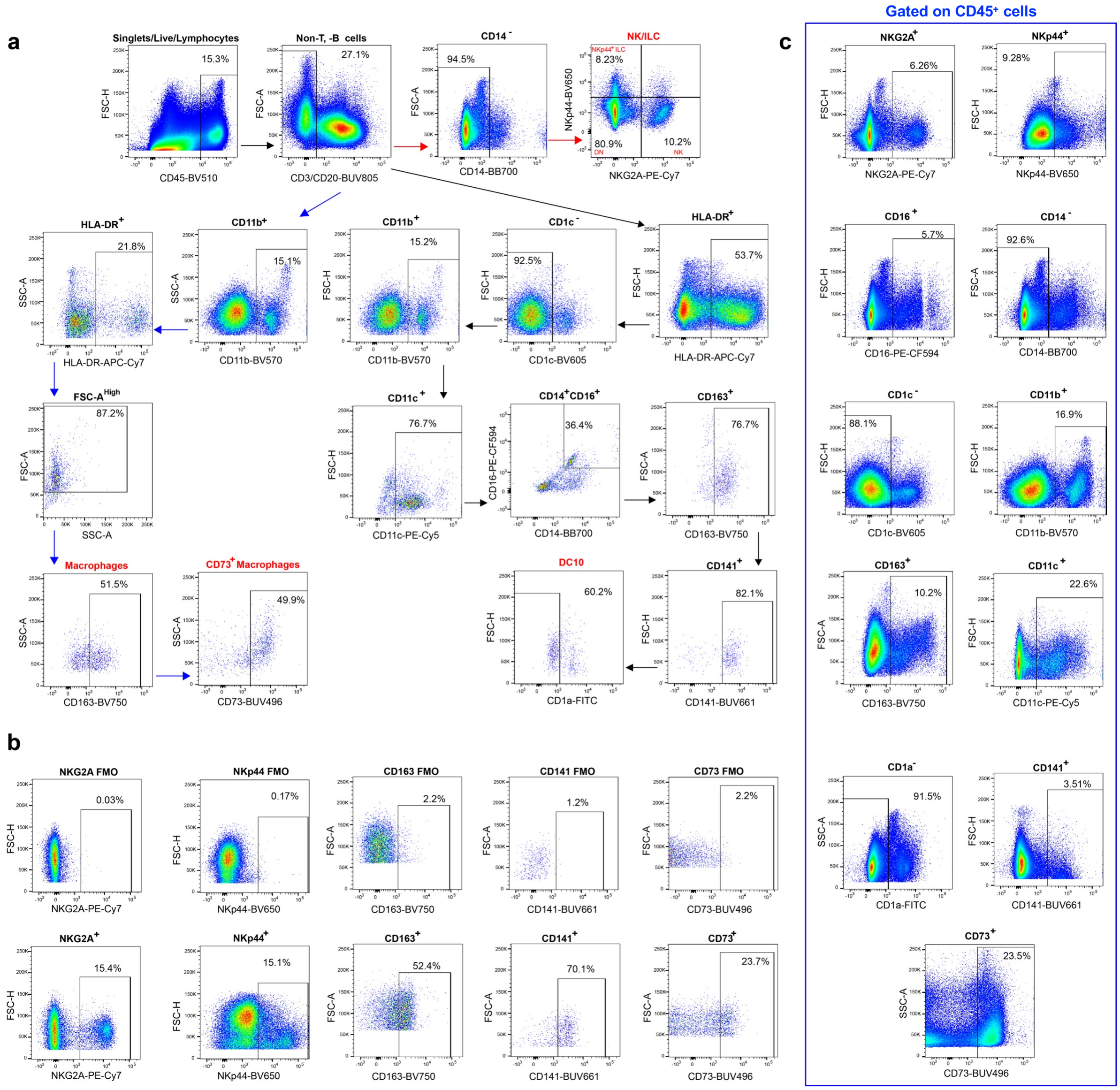

Supplementary Figure 3

Supplementary Figure 3. Representative flow plots showing the gating strategy for identifying CD73$^+$CD163$^+$ macrophages, DC-10 and innate lymphoid cells (ILCs) in rectal mucosa. (**a**) Cells were at first gated as singlets/live/CD45$^+$/CD3$^-$CD20$^-$ (non-T and -B lymphocytes). For macrophages (strategy indicated by blue arrows) cells were then gated as CD11b$^+$/HLA-DR$^+$/FSC-A^{High}SSC-A^{High}/CD163$^+$/CD73$^+$ cells and expressed as frequency of parental

population. For DC-10 (strategy indicated by black arrows) cells were gated then as HLA-DR$^+$/CD1c$^-$/CD11b$^+$/CD11c$^+$/CD14$^+$CD16$^+$/CD163$^+$/CD141$^+$/CD1a$^-$ and expressed as frequency of CD45$^+$ cells. For ILCs (strategy indicated by red arrows) cells were gated then as CD14$^-$/NKp44&NKG2A. NKG2A$^+$ NK cells were gated as NKG2A$^+$NKp44$^-$, NKp44$^+$ cells were gated as NKG2A$^-$NKp44$^+$, and NKG2A$^-$ NKp44$^-$ cells were gated as NKG2A$^-$NKp44$^-$. (**b**) Flow plots of full staining and NKG2A-PE-Cy7, NKp44-BV650, CD163-BV750, CD141-BUV661 and CD73-BUV496 Fluorescence Minus One (FMO) tubes used in the gating strategy showed in (a) and gated on their parental population. The upper row shows the FMO staining tubes, while the lower row shows the full staining tube using the same gating strategy used for the respective FMO. (**c**) Flow plots of the gating of each marker used in the gating strategy reported in (a) but directly on the live/CD45$^+$ population. This flow plots were generated to increase the number of cells present in the negative and positive populations of each marker and to facilitate the positioning of the gates in (a).

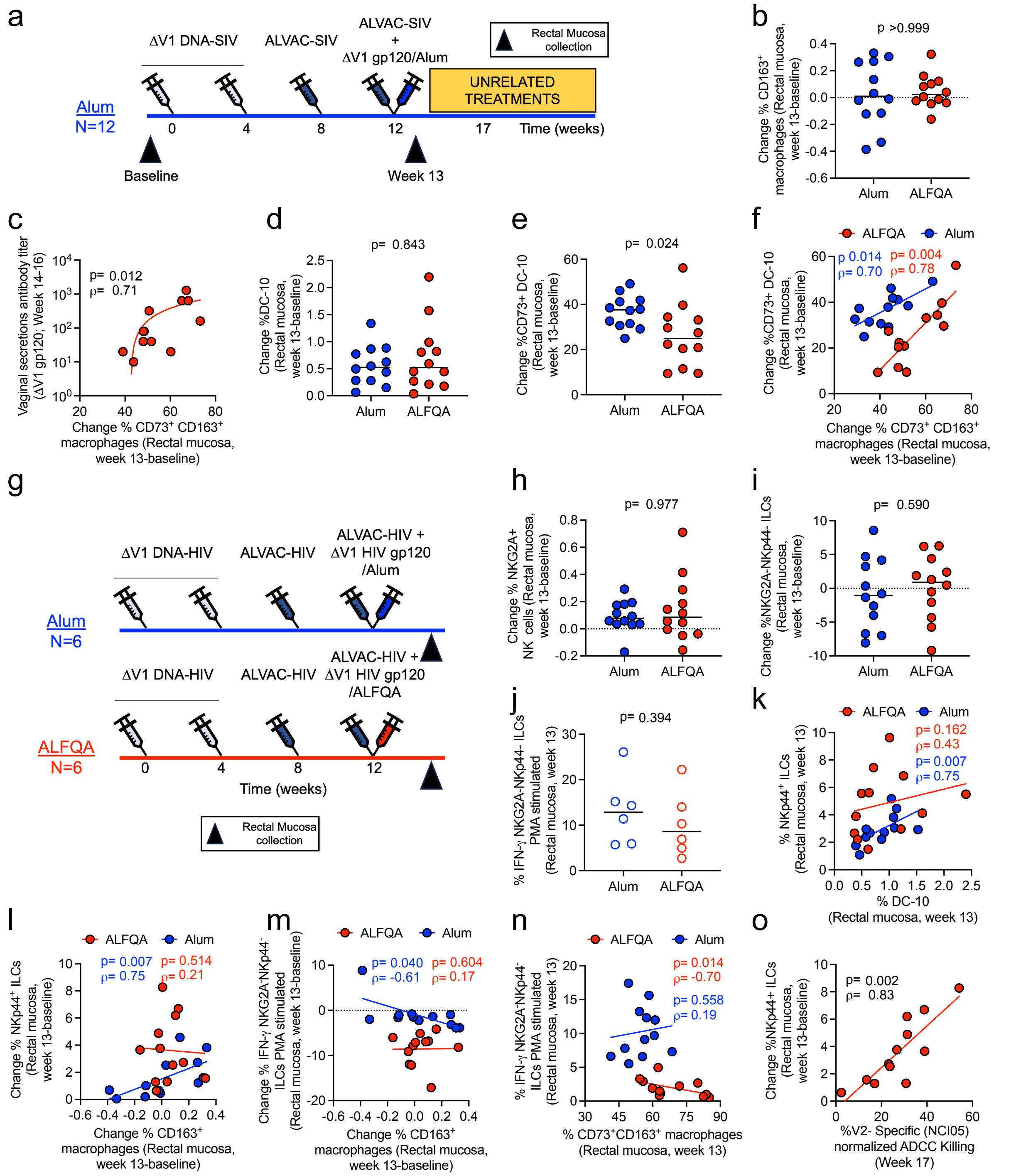

Supplementary Figure 4

Supplementary Figure 4. Mucosal vaccine-induced immune responses. (**a**) Schematic study design of Alum (blue) immunized group with immunization schedule (weeks 0–12) and collections of mucosal samples used for comparison with ALFQA samples. (**b**) Vaccine-induced

change (week 13 – baseline) of the frequency of mucosal $CD163^{+}$ macrophages in rectum of Alum (n=12) and ALFQA (n=12) animals. (**c**) Correlation between vaccine-induced change (week 13 – baseline) of the frequency of rectal $CD73^{+}CD163^{+}$ macrophages and Log_{10} vaginal antibody titers targeting the whole ΔV1gp120 protein of SIV_{m766} (weeks 14/16) in ALFQA (n=12) animals. (**d**) Vaccine-induced change (week 13 – baseline) of the frequency of mucosal DC-10 in rectum of Alum (n=12) and ALFQA (n=12) animals. (**d**) Vaccine-induced change (week 13 – baseline) of the frequency of mucosal $CD73^{+}$ DC-10 in rectum of Alum (n=12) and ALFQA (n=12) animals. (**f**) Correlation between vaccine-induced changes (week 13 – baseline) of the frequencies of rectal $CD73^{+}CD163^{+}$ macrophages and $CD73^{+}$ DC-10 of Alum (n=12) and ALFQA (n=12) animals. (**g**) Schematic study design of HIV vaccination in Alum (blue) and ALFQA (red) immunized groups with immunization schedule (weeks 0–12) and collections of mucosal samples (week 13). (**h, i**) Vaccine-induced changes (week 13 – baseline) of the frequencies of mucosal (**h**) $NKG2A^{+}$ Natural Killer cells and (**i**) $NKG2A^{-}NKp44^{-}$ ILCs in rectum of Alum (n=12) and ALFQA (n=12) animals. (**j**) Frequency of rectal IFN-γ-producing $NKG2A^{-}NKp44^{-}$ ILCs following stimulation with PMA of HIV vaccinated Alum (n=6) and ALFQA (n=6) animals (week 13). (**k**) Correlation between the frequencies of rectal $NKp44^{+}$ ILCs and DC-10 at week 13 of Alum (n=12) and ALFQA (n=12) animals. (**l, m**) Correlations between vaccine-induced changes (week 13 – baseline) in rectum of the frequencies of $CD163^{+}$ macrophages and (**l**) $NKp44^{+}$ ILCs or (**l**) IFN-γ-producing $NKG2A^{-}NKp44^{-}$ ILCs following stimulation with PMA of Alum (n=12) and ALFQA (n=12) animals. (**n**) Correlation between frequencies of rectal $CD73^{+}CD163^{+}$ macrophages and IFN-γ-producing $NKG2A^{-}NKp44^{-}$ ILCs following stimulation with PMA at week 13 of Alum (n=12) and ALFQA (n=12) animals. (**o**) Correlation between vaccine-induced changes (week 13 – baseline) in rectum of the frequency of $NKp44^{+}$ ILCs and the V2-specific ADCC (NCI05 antibody) activity against gp120 coated cells at week 14/15 in ALFQA (n=12) animals. Comparisons: (**b, d, e, h-j**) two-tailed Mann-Whitney U test with median; Correlations: (**c, f, k-o**) two-tailed Spearman correlation with simple linear regression. Alum animals and correlations are depicted in blue, ALFQA animals and correlations are depicted in red. In panels b-I and k-o, SIV-immunized Alum and ALFQA animals are depicted as black circles filled in blue and red, respectively. In panel j, HIV-immunized Alum and ALFQA animals are depicted as empty circles with blue and red borders, respectively. Source data are provided as a Source Data file.

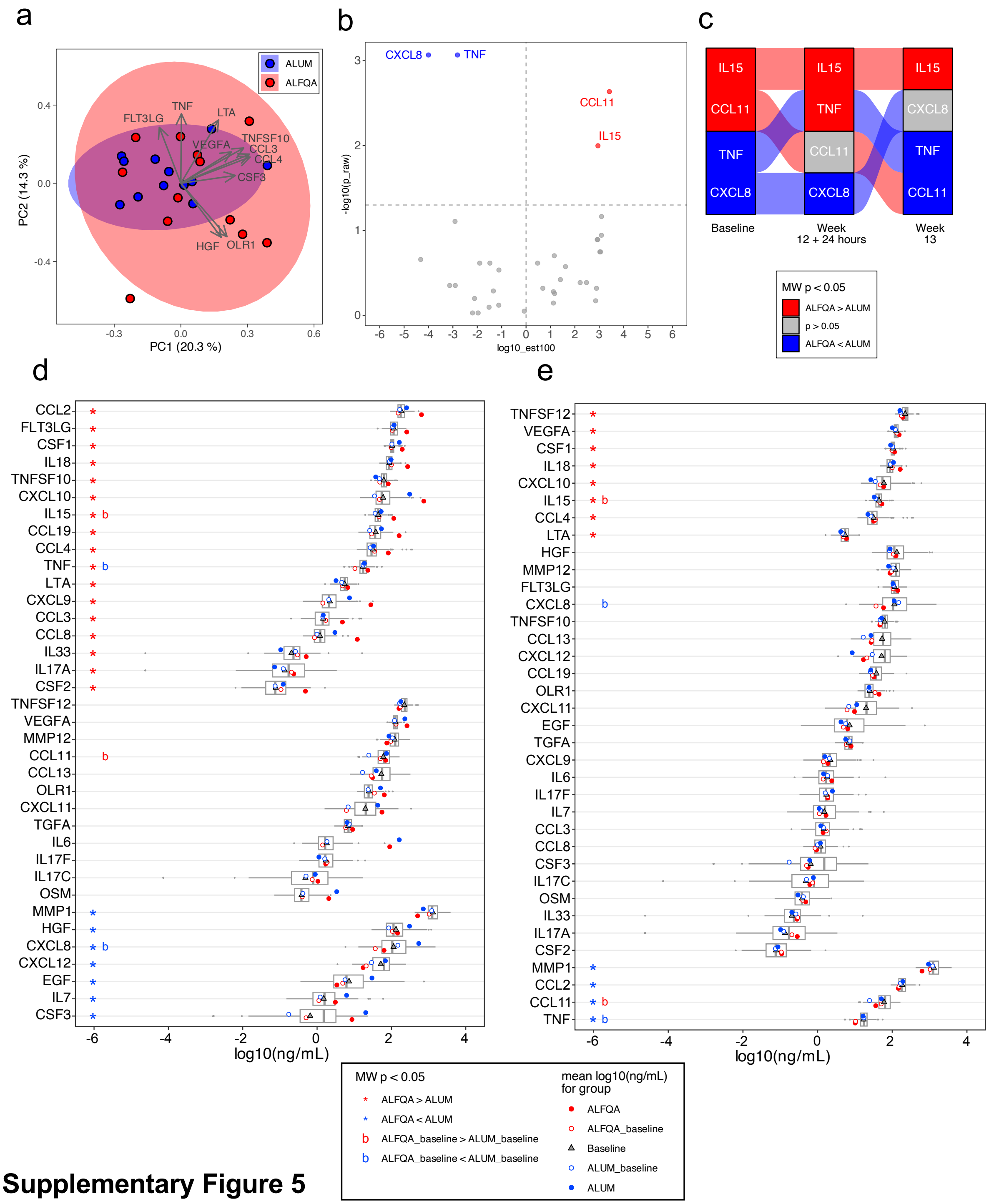

Supplementary Figure 5

Supplementary Figure 5. Vaccine-induced cytokine and chemokine milieus in Alum, ALFQA, and naïve animals. (**a**) Principal Component Analysis (PCA) plot summarizing absolute levels (pg/ml) of cytokines, chemokines, and other proteins measured in plasma collected at baseline from Alum (n=12, red circles) and ALFQA (n=12, blue circles) animals. (**b**) Volcano plots summarizing the differences in the plasma proteome at baseline in Alum (n=12) and ALFQA (n=12) animals (**c**) Alluvial diagram summarizing the pattern of Mann-Whitney significance (p<0.05) over time for plasma proteome targets that differ between the groups at baseline. At each timepoint, targets are colored according to their direction of Mann-Whitney difference p<0.05. Alluvial flow between the timepoints connect the same target at all 3 timepoints and are colored according to their pattern at baseline. (**d, e**) Summary of comparisons of absolute levels (pg/ml) of cytokines, chemokines, and other proteins measured in plasma collected at (**d**) week 12+24hours and (**e**) week 13 from Alum (blue dots; n=17 at week 12+24hours and n=12 at week 13), ALFQA (red dots; n=12 at both time-points) and naïve (gray dots; n=149 at both time-points) animals. Boxplots display the distribution of dataset by showing the first and third quartiles (25th and 75th percentiles) and the whiskers indicate 1.5*interquartile range (IQR), which represents the distance between the first and third quartiles, below the first or above the third quartile. Plotted "outliers" are data points beyond the 1.5*IQR whiskers.
Comparison between Alum and ALFQA performed by two-tailed Mann-Whitney U comparison test. Asterisk (*) indicates two-tailed Mann-Whitney U comparison test p-values between the Alum and ALFQA groups. Statistical significance: *p<0.05. Blue asterisks indicate higher values in Alum group, whereas red ones indicate higher values in ALFQA group. The letter "b" indicates significant difference (p<0.05) between baseline in Alum and ALFQA groups. Blue b indicates higher values in Alum group, whereas red b indicates higher values in ALFQA group at baseline. Source data are provided as a Source Data file.

a

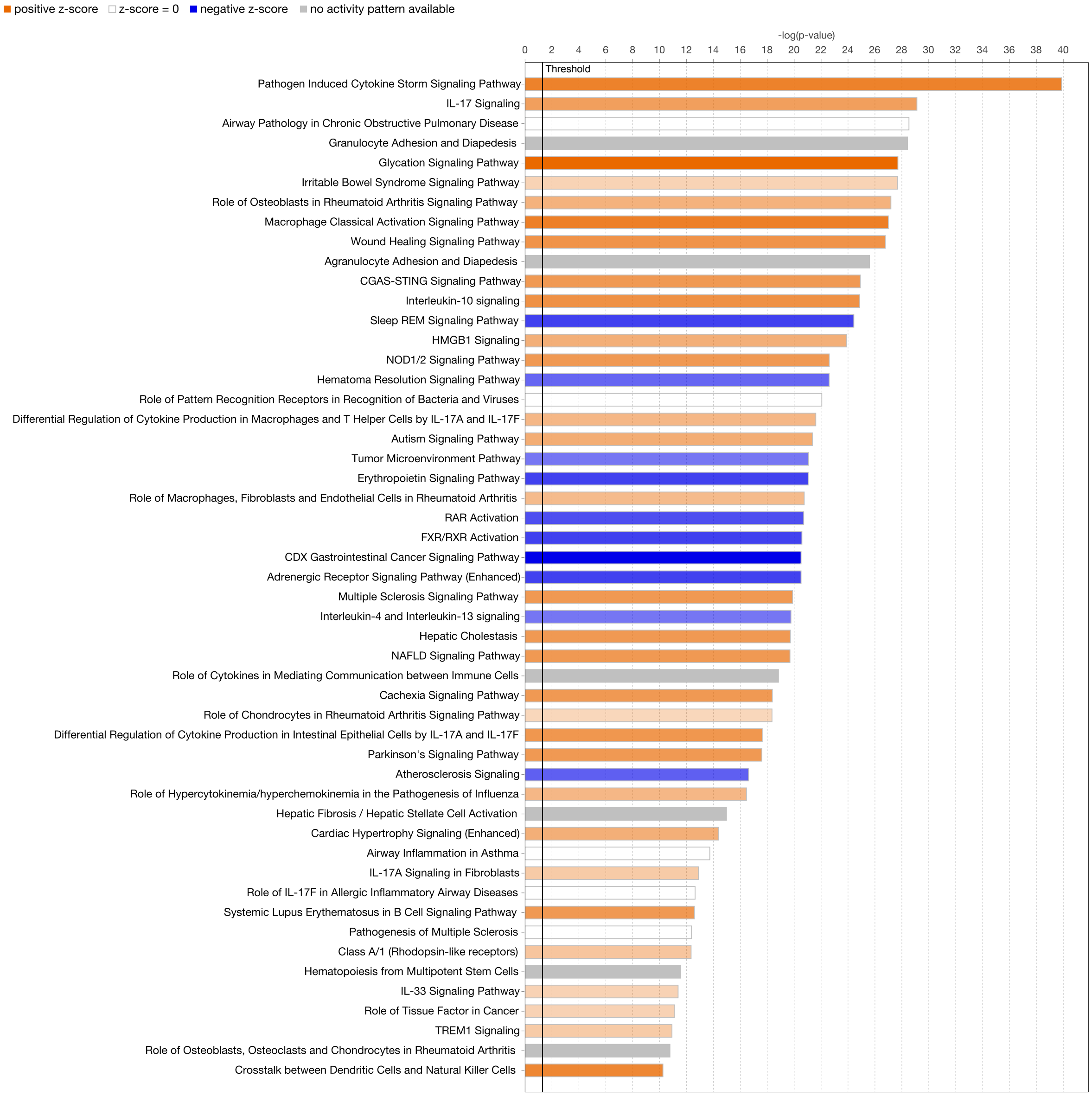

Supplementary Figure 6

b

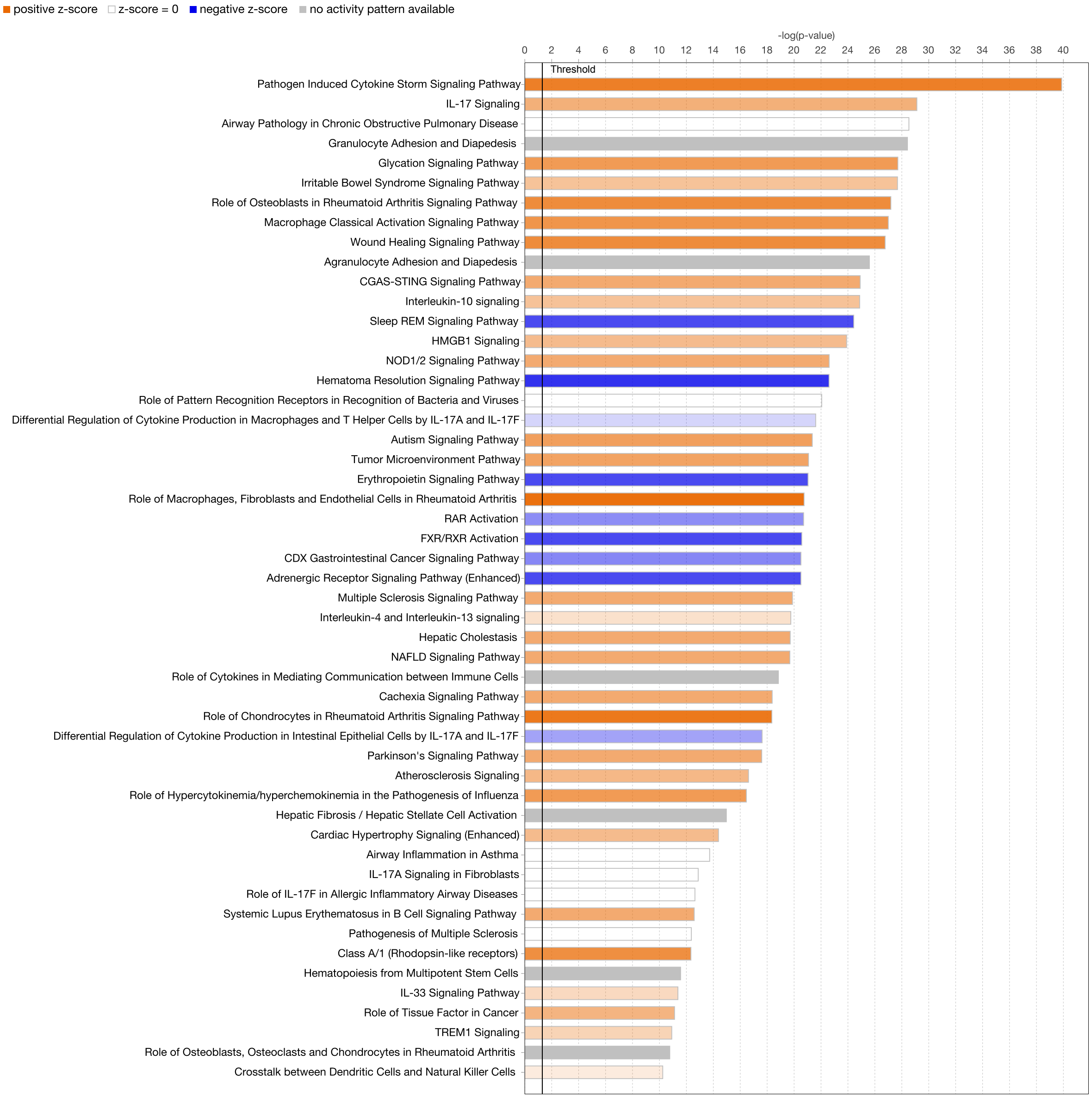

Supplementary Figure 6

Supplementary Figure 6. ALFQA-induced proteome pathways. (**a, b**) Canonical pathways induced by ALFQA proteome, compared to Alum group, identified by Ingenuity Pathway Analysis of proteome at week 12+24hours (**a**; ALFQA n=12 and Alum n=17) and week 13 (**b**; ALFQA n=12 and Alum n=12). The canonical pathways show the footprint of ALFQA on signaling and metabolic pathways. The figures show the pathways with a significant association with -log(p-value) higher than 10. P-values of overlap are calculated using the one-tailed (right) Fisher's exact test and are not adjusted for multiple comparisons. Orange and blue bars respectively indicate a positive or negative induction of the pathway by the ALFQA compared to Alum.

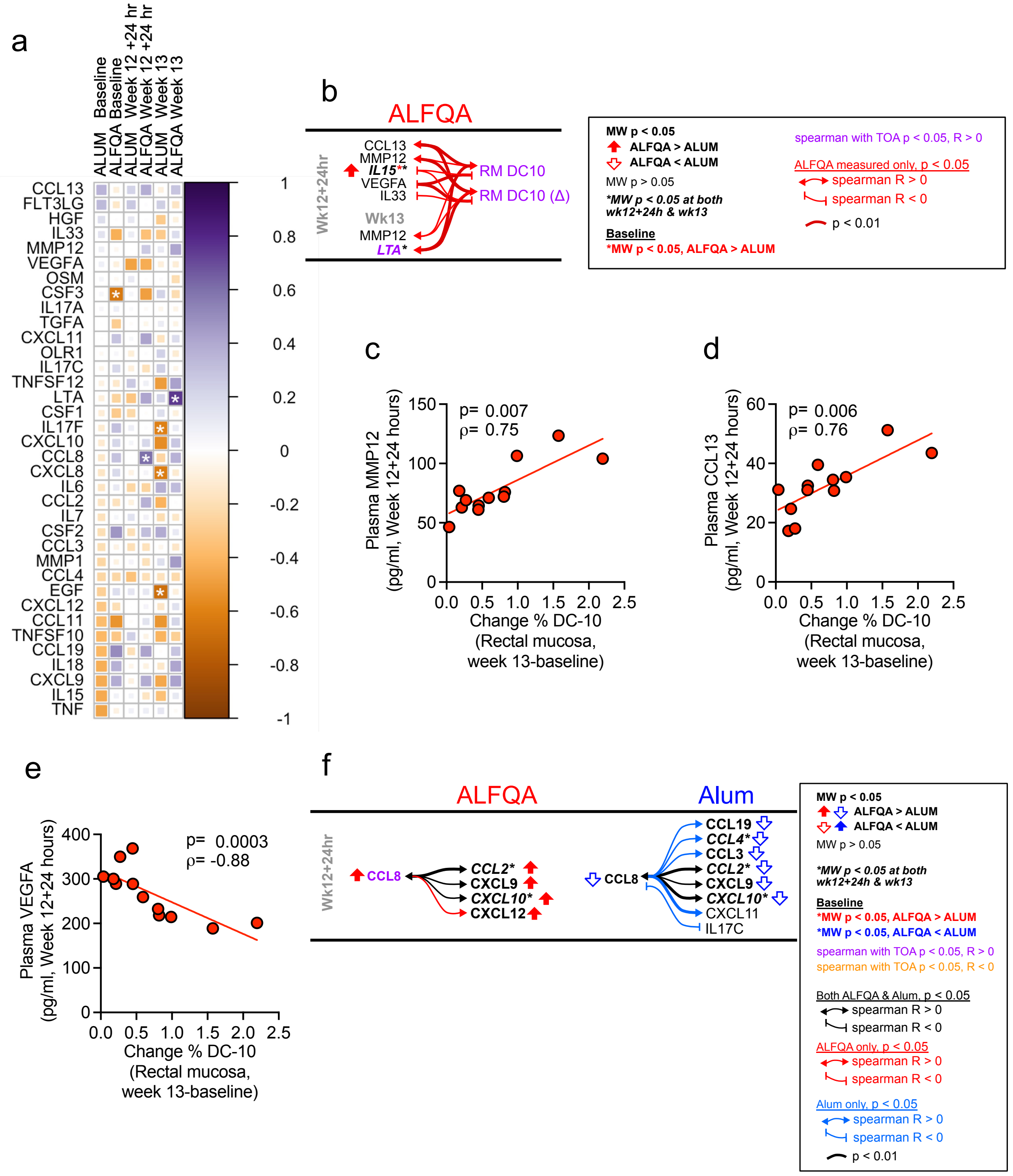

Supplementary Figure 7

Supplementary Figure 7. Cytokines and chemokines associated with risk of acquisition and influencing mucosal DC-10. (**a**) Heatmap summarizing the Spearman correlations between the absolute levels (pg/ml) of cytokines, chemokines, and other proteins measured in plasma collected at baseline from ALFQA (n=12) and Alum (n=12), and at week 12+24hours from ALFQA (n=12) and Alum (n=17), and at week 13 from ALFQA (n=12) and Alum (n=12), and time of acquisition (TOA). The ρ-values are identified by the color-scale, while the significant correlations are identified by asterisks. (**b**) Schematic summarizing two-tailed Spearman correlations between plasma biomarkers and DC-10 in the rectal mucosa, where paired samples are only available in ALFQA treated animals. Associations of $p<0.05$ shown with a thin line, and $p<0.01$ with a thicker line connecting variables. Double-headed arrows indicate spearman $R>0$, vertical stubs indicate spearman $R<0$. Mann-Whitney $p<0.05$ direction between groups is indicated by the vertical block arrows. Positive Spearman correlation with TOA $p<0.05$ is depicted with purple text. (**c-e**) Correlations between vaccine-induced change (week 13 – baseline) in rectum of the frequency of DC-10 and the plasma levels of (**c**) Matrix metalloproteinase 12 (MMP12), (**d**) C-C motif chemokine ligand 13 (CCL13) and (**e**) Vascular endothelial growth factor A (VEGFA) at week 12+24 hours in ALFQA (n=12) animals. (**f**) Schematic summarizing two-tailed Spearman correlations between TOA-associated biomarker CCL8 and other plasma biomarkers at week 12+24 hours in ALFQA (left) and Alum (right) animals. Associations of $p<0.05$ shown with a thin line, and $p<0.01$ with a thicker line connecting variables. Associations found in both Alum and ALFQA are shown in black. Associations found only in ALFQA or Alum animals are in red or blue, respectively. Double-headed arrows indicate spearman $R>0$, vertical stubs indicate spearman $R<0.05$. Mann-Whitney $p<0.05$ direction between groups is indicated by the vertical block arrows. Positive Spearman correlation with TOA $p<0.05$ is depicted with purple text. ALFQA animals and correlations are depicted as black circles filled in red and red lines.

Correlations: (**c-e**) two-tailed Spearman correlation with simple linear regression. ALFQA animals and correlations are depicted as black circles filled in in red and red lines. Source data are provided as a Source Data file.

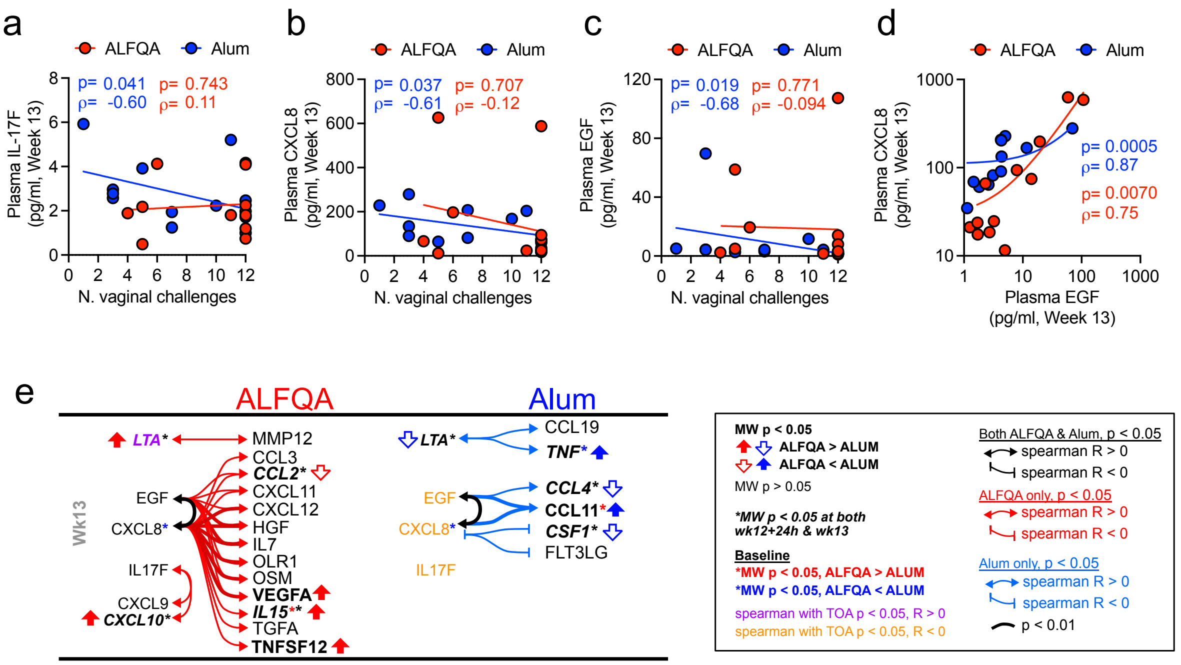

Supplementary Figure 8

Supplementary Figure 8. Cytokines and chemokines associated with increased risk of acquisition. (**a-c**) Correlations of the absolute levels (pg/ml) of plasma (**a**) Interleukin 17F (IL-17F), (**b**) C-X-C motif chemokine ligand 8 (CXCL8), or (**c**) epidermal growth factor (EGF) at week 13, with the time of acquisition (TOA) in Alum (n=12) and ALFQA (n=12) animals. (**d**) Correlation of the Log_{10} of the absolute levels (pg/ml) of plasma CXCL8 and EGF at week 13 in Alum (n=12) and ALFQA (n=12) animals. **e**) Schematic summarizing two-tailed Spearman correlations between TOA-associated biomarkers and other plasma biomarkers at week 13 in ALFQA (left) and Alum (right) animals. Associations of p<0.05 shown with a thin line, and p<0.01 with a thicker line connecting variables. Associations found in both Alum and ALFQA are shown in black. Associations found only in ALFQA or Alum animals are in red or blue, respectively. Double-headed arrows indicate spearman R>0, vertical stubs indicate spearman R<0.05. Mann-Whitney p<0.05 direction between groups is indicated by the vertical block arrows. Spearman correlation with TOA p<0.05 is depicted with purple (R>0) and orange (R<0)

text. Correlations: (**a-d**) two-tailed Spearman correlation with simple linear regression. Alum animals and correlations are depicted as black circles filled in blue and blue lines, ALFQA animals and correlations are depicted as black circles filled in red and red lines. Source data are provided as a Source Data file.

SUPPLEMENTARY TABLES

Region	20-mer peptide	Amino acid sequence
V1	15	PCVKLSPLCITMRCNKSETD
	16	PLCITMRCNKSETDRWGLTK
	17	RCNKSETDRWGLTKSSTTIT
	18	TDRWGLTKSSTTITTAAPTS
	19	TKSSTTITTAAPTSAPVSEK
	20	ITTAAPTSAPVSEKIDMVNE
	21	TSAPVSEKIDMVNETSSCIA
	22	EKIDMVNETSSCIAQNNCTG
	23	NETSSCIAQNNCTGLEQEQM
	24	IAQNNCTGLEQEQMISCKFT
V2	25	TGLEQEQMISCKFTMTGLKR
	26	QMISCKFTMTGLKRDKTKEY
	27	FTMTGLKRDKTKEYNETWYS
	28	KRDKTKEYNETWYSTDLVCE
	29	EYNETWYSTDLVCEQGNSTD

Supplementary Table 1

Supplementary Table 1. Overlapping peptides encompassing SIV V1 and V2. Amino acid sequence as single letter aa code, of the fifteen overlapping 20-mers peptides encompassing the V1 (peptides 15-24) and V2 (peptides 25-29) regions of $SIV_{M766\text{-}mac251}$.

		Alum			ALFQA			p-values Mann-Whitney U test (Alum vs ALFQA)
		Mean	Std. Deviation	Median	Mean	Std. Deviation	Median	
V1	**Peptide 15**	0.006167	0.01133	0.001	0.005583	0.006667	0.0035	undetectable response
	**Peptide 16**	0.04037	0.06104	0.006	0.005667	0.006867	0.004	undetectable response
	**Peptide 17**	0.0136	0.02386	0.004	0.03708	0.05853	0.018	undetectable response
	**Peptide 18**	0.00003333	0.0001826	0	0.003667	0.003846	0.003	undetectable response
	**Peptide 19**	0.0001667	0.0007466	0	0.003667	0.004579	0.0025	undetectable response
	**Peptide 20**	0.001	0.002816	0	0.005667	0.005646	0.005	undetectable response
	**Peptide 21**	0.0007333	0.001552	0	0.008667	0.007451	0.0085	undetectable response
	**Peptide 22**	0.01997	0.05736	0	0.146	0.2889	0.009	undetectable response
	**Peptide 23**	0.0011	0.002917	0	0.00275	0.004864	0	undetectable response
	**Peptide 24**	0.0006333	0.002327	0	0.00475	0.00477	0.003	undetectable response
V2	**Peptide 25**	0.0958	0.4262	0	0.106	0.185	0.008	undetectable response
	**Peptide 26**	1.014	0.904	0.9425	1.963	1.077	2.274	**0.016**
	**Peptide 27**	0.7428	0.7004	0.535	0.8544	0.9984	0.3905	0.9509
	**Peptide 28**	0.3325	0.4176	0.1675	0.1213	0.1474	0.03	0.115
	**Peptide 29**	0.008067	0.01234	0.0045	0	0	0	undetectable response

Supplementary Table 2

Supplementary Table 2. Antibody responses to overlapping peptides encompassing SIV V1 and V2. Serum antibody responses to overlapping peptides encompassing the V1 and V2 of $SIV_{M766-mac251}$. Serum from Alum (n=30) and ALFQA (n=12) animals collected at weeks 14-17 was evaluated by Pepscan ELISA. The data represented show the mean, standard deviation, and median of the optical density (OD) of the antibody responses. The last column shows the unadjusted p-values of the comparisons between the two groups using two-tailed Mann-Whitney test. Comparisons between undetectable responses (mean OD < 0.05) were not performed. Source data are provided as a Source Data file.

		Neutralization ID_{50}				Neutralization ID_{80}			
		TIER 1A SIVmac251.6 (ID#1636DB2)	TIER 1A SIVsmE660 /BR-CG7G.IR1 (ID#1370DB2)	**TIER 1B SIVsmE660 /BR-CG7V.IR1 (ID#1634DB2)**	**Challenge virus SIVmac251 DAY 8 2010 (ID#2079DB2)**	**TIER 1A SIVmac251.6 (ID#1636DB2)**	TIER 1A SIVsmE660 /BR-CG7G.IR1 (ID#1370DB2)	TIER 1B SIVsmE660 /BR-CG7V.IR1 (ID#1634DB2)	**Challenge virus SIVmac251 DAY 8 2010 (ID#2079DB2)**
Alum	25% Percentile	50846	4107	**784.3**	**145**	**1602**	1045	20	**23.25**
	Median	143549	14340	**2465**	**176.5**	**4725**	4109	20	**30**
	75% Percentile	570026	50800	**11293**	**221.3**	**12954**	14033	20	**34.75**
	95% CI	50474-680502	3126-51964	**607-13543**	**139-223**	**1375-14748**	725-14159	20-20	**23-35**
ALFQA	25% Percentile	11110	365.3	**42**	**55.75**	**200**	200	20	**20**
	Median	44355	1705	**104**	**67.5**	**200**	625	20	**20**
	75% Percentile	190183	26408	**617.8**	**152.3**	**200**	6595	20	**20**
	95% CI	10602-227977	360-30061	**41-619**	**54-170**	**200-200**	200-7356	20-20	**20-20**
Alum vs ALFQA	Mann-Whitney Test P-value	0.0582	0.0866	**0.0009**	**0.0014**	**0.0000007**	0.1544	>0.9999	**0.000010**
Spearman Correlation Alum	ρ-value	0.16	0.17	0.039	**-0.62**	0.3	0.085	NA	-0.58
	p-value	0.609	0.601	0.907	**0.035**	0.342	0.792	NA	0.05
Spearman Correlation ALFQA	ρ-value	-0.031	-0.31	-0.36	-0.074	0.24	-0.31	NA	NA
	p-value	0.926	0.317	0.244	0.82	0.917	0.319	NA	NA

Supplementary Table 3

Supplementary Table 3. Plasma neutralizing antibody responses to SIV viruses and correlation with risk of infection. The data represented show the median, the 25th and 75th percentiles, and the 95% Confidence Intervals (CI) of Median Infectious Dose 50 (ID_{50}) and 80 (ID_{80}) neutralizing antibody responses to different SIV viruses (TIER 1A/SIV_{mac251}, TIER 1A/SIV_{smE660}, TIER 1B/SIV_{smE660} and SIV_{mac251} used for the animal challenge) in plasma collected at week 14 in Alum (n=12) and week 15 in ALFQA (n=12) animals. The "Alum vs ALFQA" row shows the unadjusted p-values of the comparisons between the two groups using two-tailed Mann-Whitney test. Cells highlighted in blue indicate a higher response in Alum vs ALFQA animals. The "Spearman correlation Alum" and "Spearman correlation ALFQA" rows show two-tailed Spearman correlation coefficients (ρ) and unadjusted p-values for the association between the neutralizing antibodies titers and the risk of infection. Significant differences and correlations are indicated in bold. Source data are provided as a Source Data file.

	Week 12 + 24 hours						p-values Mann-Whitney U test (Alum vs ALFQA)
	Alum			ALFQA			
	Mean	Std. Deviation	Median	Mean	Std. Deviation	Median	
**CCL8**	3.77	2.989	2.838	13.97	7.458	12.52	0.00002
**IL33**	0.1343	0.08877	0.148	0.729	0.7395	0.5222	0.00001
**CXCL12**	73.53	25.49	71.85	21.2	13.52	17.71	0.000001
**OLR1**	52.66	15.42	45.57	68.15	24.08	62.98	0.053
**IL27**	Not reliable	Not reliable	Not reliable	Not reliable	Not reliable	Not reliable	NA
**IL2**	Not reliable	Not reliable	Not reliable	Not reliable	Not reliable	Not reliable	NA
**CXCL9**	9.167	6.323	6.923	39.43	42.11	22.48	0.00006
**TGFA**	6.625	1.854	6.734	10.03	5.389	7.865	0.088
**IL1B**	Not reliable	Not reliable	Not reliable	Not reliable	Not reliable	Not reliable	NA
**IL6**	279	298.9	140.2	110.1	78.24	76.45	0.152
**IL4**	Not reliable	Not reliable	Not reliable	Not reliable	Not reliable	Not reliable	NA
**TNFSF12**	179.9	25.96	178.5	160.8	23.1	167.3	0.059
**TSLP**	Not reliable	Not reliable	Not reliable	Not reliable	Not reliable	Not reliable	NA
**CCL11**	77.56	25.07	73.38	73.43	23.56	70.55	0.679
**HGF**	361.6	221	333.2	200.8	184.9	137.2	0.012
**FLT3LG**	125.1	46.9	119	281.4	148.5	229.8	0.000004
**IL17F**	1.456	1.25	1.093	1.917	0.9806	1.531	0.097
**IL7**	7.689	5.135	5.533	4.376	4.607	2.887	0.030
**IL13**	Not reliable	Not reliable	Not reliable	Not reliable	Not reliable	Not reliable	NA
**IL18**	101.4	30.49	100.6	280.1	63.99	279.8	0.00000008
**CCL13**	58	54.47	35.42	32.45	9.749	31.79	0.616
**TNFSF10**	40.14	14.47	39.98	84.78	24.56	78.59	0.000003
**CXCL10**	369.2	227.5	334.2	786.6	261.9	763.7	0.0001
**IFNG**	Not reliable	Not reliable	Not reliable	Not reliable	Not reliable	Not reliable	NA
**IL10**	Not reliable	Not reliable	Not reliable	Not reliable	Not reliable	Not reliable	NA
**CCL19**	58	22.23	49.29	186	101.5	164.6	0.000008
**TNF**	20.61	11.22	17.29	23.44	3.887	22.28	0.011
**IL15**	53.71	9.897	53.01	119.3	30.76	111.9	0.00000004
**CCL3**	1.723	1.284	1.215	5.853	5.29	3.95	0.00002
**CXCL8**	780.8	574.2	732.7	81.99	54.07	71.52	0.0000007
**MMP12**	89.77	26.59	85.84	77.82	22.2	71.57	0.195
**CSF2**	0.2142	0.2312	0.1239	0.5669	0.2718	0.507	0.0009
**CSF3**	27.87	21.15	24.67	11.8	8.033	11.2	0.018
**VEGFA**	233.1	42.81	229.8	268	58.45	274.1	0.152
**IL17C**	1.434	1.462	0.7671	3.079	5.919	1.463	0.845
**EGF**	45	41.34	36.29	4.323	2.952	4.132	0.0000005
**CCL2**	270.3	95.48	233.6	688.8	285.1	584.2	0.0000003
**IL17A**	0.2182	0.3655	0.05501	0.4883	0.5906	0.2723	0.034
**OSM**	3.886	2.117	3.301	2.555	1.591	2.233	0.073
**CSF1**	163.8	17.51	159.1	196.3	22.59	197.9	0.0002
**CCL4**	36.11	19.92	29.02	103.9	108.9	68.46	0.00001
**CXCL11**	55.5	46.03	42.65	65.71	41.71	55.36	0.347
**LTA**	3.397	1.32	3.124	7.133	2.623	6.616	0.000005
**CCL7**	Not reliable	Not reliable	Not reliable	Not reliable	Not reliable	Not reliable	NA
**MMP1**	776.5	325.2	690	522.3	124.2	524.7	0.012

Supplementary Table 4

Supplementary Table 4. Cytokine/chemokine milieu induced by vaccinations at week 12+24 hours. The data represented show the mean, standard deviation, and median of the absolute levels (pg/ml) of 48 biomarkers measured by proximity extension assay in plasma collected at week 12+24 hours in Alum (n=17) and ALFQA (n=12) animals. The last column shows the unadjusted p-values of the comparisons between the two groups using two-tailed Mann-Whitney test. Source data are provided as a Source Data file.

	Week 13						p-values Mann-Whitney U test (Alum vs ALFQA)
	Alum			ALFQA			
	Mean	Std. Deviation	Median	Mean	Std. Deviation	Median	
**CCL8**	1.241	0.375	1.136	1.022	0.4206	1.061	0.242
**IL33**	0.2259	0.1316	0.1808	0.3263	0.2262	0.2239	0.060
**CXCL12**	20.49	14.21	19.13	32.93	30.23	26.99	0.410
**OLR1**	25.63	10.82	22.03	116.5	202.3	32.8	0.101
**IL27**	Not reliable	Not reliable	Not reliable	Not reliable	Not reliable	Not reliable	NA
**IL2**	Not reliable	Not reliable	Not reliable	Not reliable	Not reliable	Not reliable	NA
**CXCL9**	1.717	0.8559	1.492	2.132	1.029	2.303	0.347
**TGFA**	5.948	2.134	5.383	8.788	4.992	7.386	0.052
**IL1B**	Not reliable	Not reliable	Not reliable	Not reliable	Not reliable	Not reliable	NA
**IL6**	1.662	1.042	1.369	3.308	3.139	1.781	0.114
**IL4**	Not reliable	Not reliable	Not reliable	Not reliable	Not reliable	Not reliable	NA
**TNFSF12**	162.3	26.99	155.4	196.9	44.72	199	0.028
**TSLP**	Not reliable	Not reliable	Not reliable	Not reliable	Not reliable	Not reliable	NA
**CCL11**	52.84	14	53.11	37.14	9.118	36.87	0.010
**HGF**	108	80.24	68.74	203.4	212.2	95.38	0.443
**FLT3LG**	110.1	34.46	111	145.9	45.83	145	0.089
**IL17F**	2.828	1.503	2.517	2.225	1.266	1.933	0.291
**IL7**	1.532	1.078	1.624	3.482	5.767	0.9963	0.347
**IL13**	Not reliable	Not reliable	Not reliable	Not reliable	Not reliable	Not reliable	NA
**IL18**	112	39.55	96.92	169.2	44.37	163.9	0.005
**CCL13**	29.32	12.54	24.7	30.55	14.81	32.01	0.977
**TNFSF10**	53.49	13.62	52.46	47.78	11.84	47.23	0.378
**CXCL10**	28.09	9.495	28.09	65.48	29.78	57.31	0.001
**IFNG**	Not reliable	Not reliable	Not reliable	Not reliable	Not reliable	Not reliable	NA
**IL10**	Not reliable	Not reliable	Not reliable	Not reliable	Not reliable	Not reliable	NA
**CCL19**	27.4	6.863	28.38	35.97	16.94	30.25	0.291
**TNF**	16.92	3.911	17.39	10.83	3.94	10.79	0.001
**IL15**	34.45	12.51	31.73	57.99	30.81	47.32	0.002
**CCL3**	1.329	0.6111	1.155	1.884	2.081	1.344	0.755
**CXCL8**	135	79.82	112	146.9	221.2	45.48	0.128
**MMP12**	84.21	28.98	75.26	91.6	30.9	94.23	0.671
**CSF2**	0.2292	0.368	0.06849	0.1816	0.2295	0.1041	0.478
**CSF3**	2.661	2.985	1.33	2.41	3.738	1.488	>0.999
**VEGFA**	100.9	7.725	101.2	161.2	57.56	149.1	0.0002
**IL17C**	1.214	0.9664	0.9849	3.051	7.382	1.042	0.671
**EGF**	9.463	19.14	4.245	18.78	32.25	4.096	0.630
**CCL2**	196.9	43.13	201.1	154.2	56.65	137.1	0.028
**IL17A**	0.2741	0.4134	0.09808	0.5077	0.4902	0.2591	0.143
**OSM**	0.3523	0.1954	0.3283	0.7534	0.9766	0.454	0.347
**CSF1**	91.08	13.21	87.56	117.2	20.04	120.2	0.002
**CCL4**	23.3	8.01	21.65	35.93	23.99	28.58	0.039
**CXCL11**	19.32	24.24	6.925	20.52	29.22	7.724	0.478
**LTA**	4.229	0.7713	4.24	6.135	1.529	5.72	0.001
**CCL7**	Not reliable	Not reliable	Not reliable	Not reliable	Not reliable	Not reliable	NA
**MMP1**	974.9	297.7	942.4	663.3	227.6	637	0.012

Supplementary Table 5

Supplementary Table 5. Cytokine/chemokine milieu induced by vaccinations at week 13. The data represented show the mean, standard deviation, and median of the absolute levels (pg/ml) of 48 biomarkers measured by proximity extension assay in plasma collected at week 13 in Alum (n=12) and ALFQA (n=12) animals. The last column shows the unadjusted p-values of the comparisons between the two groups using two-tailed Mann-Whitney test. Source data are provided as a Source Data file.
