# Supplementary material for: HIV vaccine candidate ΔV1gp120 formulated in ALFQA adjuvant augments mucosal immunity in female macaques
Source: Nat Commun. 2025 Sep 29;16:8571. doi: 10.1038/s41467-025-63610-z (PMC12480912; doi:10.1038/s41467-025-63610-z)
Supplement: Supplementary file 2 — Description of Additional Supplementary File [file 41467_2025_63610_MOESM2_ESM.pdf]

Description of Additional Supplementary File

Supplementary Data 1: Unadjusted p and r (R) values of the two-tailed Spearman correlations between the levels (pg/ml) of each plasma proteome target separated per group (See excel file). The table reports the timepoints and the groups at which the targets for correlations were measured, the targets used for each correlation (Targets 1 and 2), the Spearman r (R), and the Spearman p-values unadjusted for multiple comparisons. Source data are provided as a Source Data file.

Supplementary Data 2: Pathways identified by Ingenuity Pathway Analysis of the ALFQAinduced proteome at week 12+24hours (ALFQA n=12 and Alum n=17) (See excel file). The table reports the names of the pathways identified, ranked by their significance [-log(p-value)], with their z-score, indicating their activation or inhibition, and the molecules present in the proteome that are included in each pathway. P-values of overlap are calculated using the onetailed (right) Fisher's exact test and are not adjusted for multiple comparisons. Source data are provided as a Source Data file

Supplementary Data 3**:** Pathways identified by Ingenuity Pathway Analysis of the ALFQAinduced proteome at week 13 (ALFQA n=12 and Alum n=12) (See excel file). The table reports the names of the pathways identified, ranked by their significance [-log(p-value)], with their zscore, indicating their activation or inhibition, and the molecules present in the proteome that are included in each pathway. P-values of overlap are calculated using the one-tailed (right) Fisher's exact test and are not adjusted for multiple comparisons. Source data are provided as a Source Data file.
