# Supplementary material for: HIV vaccine candidate ΔV1gp120 formulated in ALFQA adjuvant augments mucosal immunity in female macaques
Source: Nat Commun. 2025 Sep 29;16:8571. doi: 10.1038/s41467-025-63610-z (PMC12480912; doi:10.1038/s41467-025-63610-z)
Supplement: Supplementary file 6 — Reporting summary [file 41467_2025_63610_MOESM6_ESM.pdf]

nature portfolio

Corresponding author(s): Genoveffa Franchini, Massimiliano Bissa

Last updated by author(s): Jun 30, 2025

Reporting Summary

Nature Portfolio wishes to improve the reproducibility of the work that we publish. This form provides structure for consistency and transparency in reporting. For further information on Nature Portfolio policies, see our Editorial Policies and the Editorial Policy Checklist.

Statistics

For all statistical analyses, confirm that the following items are present in the figure legend, table legend, main text, or Methods section.

n/a	Confirmed	
☐	☒	The exact sample size (*n*) for each experimental group/condition, given as a discrete number and unit of measurement
☐	☒	A statement on whether measurements were taken from distinct samples or whether the same sample was measured repeatedly
☐	☒	The statistical test(s) used AND whether they are one- or two-sided<br>*Only common tests should be described solely by name; describe more complex techniques in the Methods section.*
☐	☒	A description of all covariates tested
☐	☒	A description of any assumptions or corrections, such as tests of normality and adjustment for multiple comparisons
☐	☒	A full description of the statistical parameters including central tendency (e.g. means) or other basic estimates (e.g. regression coefficient) AND variation (e.g. standard deviation) or associated estimates of uncertainty (e.g. confidence intervals)
☐	☒	For null hypothesis testing, the test statistic (e.g. *F*, *t*, *r*) with confidence intervals, effect sizes, degrees of freedom and *P* value noted<br>*Give P values as exact values whenever suitable.*
☒	☐	For Bayesian analysis, information on the choice of priors and Markov chain Monte Carlo settings
☒	☐	For hierarchical and complex designs, identification of the appropriate level for tests and full reporting of outcomes
☒	☐	Estimates of effect sizes (e.g. Cohen's *d*, Pearson's *r*), indicating how they were calculated

Our web collection on statistics for biologists contains articles on many of the points above.

Software and code

Policy information about availability of computer code

Data collection	no software was used
Data analysis	GraphPad Prism (Version 10.3.0 (461)) was used to calculate the statistics.<br><br>Flow cytometry data were analyzed using FlowJo v10.1 (TreeStar, Inc.) and FlowJo version 10.6<br><br>Proteome data validation to uphold data integrity was conducted with the Olink NPX Signature software (Olink Proteomics AB, Uppsala, Sweden), specifically designed for the Olink® analysis: the application was used to import data from the Olink Signature Q100 instrument and process the data.<br><br>Qiagen Ingenuity Pathway Analysis application (Version 01-23-01; Qiagen Sciences, Germantown, MD, USA) was used to perfume pathway analysis.<br><br>R version 4.4.1<br>ggalluvial_0.12.5 – for alluvial diagrams<br>corrplot_0.95 – for correlation heatmaps (supp fig 7a) ggpubr_0.6.0 – other plots<br>The source codes for the analyses are available at: https://github.com/NCI-VB/franchini_bissa_ALFQAvsAlum, and can be cited using DOI https://doi.org/10.5281/zenodo.15747628.

For manuscripts utilizing custom algorithms or software that are central to the research but not yet described in published literature, software must be made available to editors and reviewers. We strongly encourage code deposition in a community repository (e.g. GitHub). See the Nature Portfolio guidelines for submitting code & software for further information.

Data

Policy information about availability of data

All manuscripts must include a data availability statement. This statement should provide the following information, where applicable:

- Accession codes, unique identifiers, or web links for publicly available datasets
- A description of any restrictions on data availability
- For clinical datasets or third party data, please ensure that the statement adheres to our policy

Source Data are provided with this manuscript in the Source Data file. The original data and files used to generate the data presented in this study have been deposited in the Zenodo repository (https://zenodo.org/) under accession code (DOI 10.5281/zenodo.15602857)

Research involving human participants, their data, or biological material

Policy information about studies with human participants or human data. See also policy information about sex, gender (identity/presentation), and sexual orientation and race, ethnicity and racism.

Reporting on sex and gender	Not Applicable
Reporting on race, ethnicity, or other socially relevant groupings	Not Applicable
Population characteristics	Not Applicable
Recruitment	Not Applicable
Ethics oversight	Not Applicable

Note that full information on the approval of the study protocol must also be provided in the manuscript.

Field-specific reporting

Please select the one below that is the best fit for your research. If you are not sure, read the appropriate sections before making your selection.

☒ Life sciences ☐ Behavioural & social sciences ☐ Ecological, evolutionary & environmental sciences

For a reference copy of the document with all sections, see nature.com/documents/nr-reporting-summary-flat.pdf

Life sciences study design

All studies must disclose on these points even when the disclosure is negative.

Sample size

Based on these previous results, we consider several different situations where the infection rates in DNA/ALVAC/gp120/ALFQA vaccine group are similar with and lower than the rates in the control. Table 1 shows the power calculations of three different infection rates in the vaccine group of 12 sample size, given the historical control group of 37 sample size, using a log rank test with a significance level of 0.05.

Table 1. Power calculations with different infection rates in DNA/ALVAC/gp120/ALFQA

Infection rates in DNA/ALVAC/gp120/ALFQA	0.108	0.1	0.08	0.07	0.065
Power*	0.4383	0.4994	0.6979	0.8061	0.8540

the power calculations are based on 10,000 simulated datasets where the infection rate in the control is 0.22.

For instance, with the total sample size of 49 (12 in the vaccination and 37 in the control), there would be 80.6% power to detect a difference in virus acquisition, given the infection rates of 7% and 22% in the vaccine and control groups, respectively. Overall, the power would be expected in a various range of between about 44% and 85% depending on infection rates of the vaccine group in Table 1.

Data exclusions

Data were not excluded from the analyses

Replication

The nature of the samples analyzed in the present studies, the limited amount of each sample collected from each animal and the cost of the non-human primate studies do not allow us to replicate the experiments. In the reported assays the replicates are represented by each animal enrolled in the study. All the data have been obtained with validated assays that have been used in previous publish work

Randomization

The macaques were assigned to groups based on their age, sex and weight.

Blinding

For macaques studies (immunizations and viral exposures): the investigators that prepared the reagents were unblinded whereas the veterinarian staff was blinded.
For immunological assays: during the execution and analyses of the experiments the investigators were blinded. Investigators were unblinded after generating the results in order to perform the final analyses of the data and correlation studies.

Reporting for specific materials, systems and methods

We require information from authors about some types of materials, experimental systems and methods used in many studies. Here, indicate whether each material, system or method listed is relevant to your study. If you are not sure if a list item applies to your research, read the appropriate section before selecting a response.

Materials & experimental systems

n/a	Involved in the study
☐	☒ Antibodies
☐	☒ Eukaryotic cell lines
☒	☐ Palaeontology and archaeology
☐	☒ Animals and other organisms
☒	☐ Clinical data
☒	☐ Dual use research of concern
☒	☐ Plants

Methods

n/a	Involved in the study
☒	☐ ChIP-seq
☐	☒ Flow cytometry
☒	☐ MRI-based neuroimaging

Antibodies

Antibodies used

As reported in the Method section:
Trogocytosis: APC-H7-conjugated anti-CD14 antibody (5ul, clone MΦP9, Cat# 560180, BD Biosciences).
ADNP: Staining was conducted using Anti-human CD3 AF700 (5ul, clone UCHT1, Cat# 557943) and anti-human CD14 APC-Cy7 (5ul, clone MΦP9, Cat# 557831) antibodies obtained from BD Biosciences, and anti-human CD66b Pacific Blue (5ul, clone G10F5, Cat# 305112) antibody from BioLegend.

Flow cytometry of rectal mucosal cells:CCells were stained with Live/Dead blue dye (cat. #L34962, 0.5 µl) from Thermo Fisher, followed by surface staining for 30 minutes at RT with the following antibodies: BB700 anti-CD14 (M5E2; cat. # 745790, 5µl), APC anti-CCR2 (48607; cat. # 558406, 5µl), APC-Cy7 anti-HLA-DR (L243; cat. # 335796, 5µl), BV480 anti-CD45 (D058-1283; cat. # 566145, 5µl), BV650 anti-NKp44 (p44-8; cat. # 744302, 5µl), BV750 anti-CD163 (GHI/61; cat. # 747185, 5µl), BUV493 anti-CD73 (AD2; cat. # 750061, 5µl), BUV563 anti-CD184(CXCR4) (12G5; cat. # 741400, 5µl), BUV661 anti-CD141 (1A4; cat. # 741650, 5µl), BUV737 anti-CD206 (19.2; cat. # 741860, 5µl), BUV805 anti-CD3 (SP34-2; cat. # 742053, 5µl), BUV805 anti-CD20 (2H7; cat. # 612905, 5µl) from BD Biosciences (San Jose, California, USA); PE-Cy7 anti-NKG2A (Z199; cat. no. B10246, 5 µl) from Beckman Coulter (Brea, California, USA); AF488 anti-CD1a (O10; cat. No. NBP2-34697AF488, 5 µl) from Novus Biologicals (Centennial, Colorado, USA); PE anti-CD33 (AC104.3E3; cat. #130-113-349, 5µl) from Miltenyi Biotec (Bergisch Gladbach, North Rhine-Westphalia, Germany); and PE/Dazzle594 anti-CD16 (3G8; cat. # 302054, 5µl), PE-Cy5 anti-CD11c (3.9; cat. # 301610, 5µl), BV570 anti-CD11b (ICRF44; cat. # 301325, 5µl), BV605 anti-CD1c (L161; cat. # 331538, 5µl) from BioLegend (San Diego, California, USA).

Cytokine expression upon stimulation of innate lymphoid cells in rectal mucosa:cells were stained with Live/Dead blue dye (cat. #L34962, 0.5 µl) from Thermo Fisher, followed by surface staining for 30 minutes at room temperature. Surface staining was conducted as described above for phenotyping of innate lymphoid, myeloid and dendritic cells in rectal mucosa. Following surface staining, cells were fixed and permeabilized with a FOX3-transcription buffer set (cat. #00-5523-00) from eBioscience (San Diego, California, USA) according to the manufacturer's recommendation and subsequently intracellular staining with the following: R718 anti-TNF? (MAb11; cat. #566957, 5µl), BV711 anti-IL-10 (JES3-9D7; cat. # 564050, 5µl), BV786 anti-CD107 (H4A3; cat. # 563869, 5µl), BUV395 anti-IFN-γ (B27; cat. # 563563, 5µl) from BD Biosciences (San Jose, California, USA); and BV421 anti-IL-17 (BL168; cat. # 512312, 5µl) from BioLegend (San Diego, California, USA).

Validation

All the antibodies used are reported as reactive with human or rhesus macaque cells.
Reactivity with human cells
anti-CD14 antibody clone MΦP9, https://www.bdbiosciences.com/en-us/products/reagents/flow-cytometry-reagents/research-reagents/single-color-antibodies-ruo/apc-h7-mouse-anti-human-cd14.560180?tab=product_details;
Anti-human CD3 clone UCHT1, https://www.bdbiosciences.com/en-us/products/reagents/flow-cytometry-reagents/research-reagents/single-color-antibodies-ruo/alexa-fluor-700-mouse-anti-human-cd3.557943?tab=product_details;
anti-human CD66b clone G10F5, https://www.biolegend.com/en-gb/products/pacific-blue-anti-human-cd66b-antibody-9583;

Reactivity with Rhesus macaque cells
anti-CD14 clone M5E2, https://www.nhpreagents.org/ReactivityDatabase;
anti-CCR2 clone 48607, https://www.nhpreagents.org/ReactivityDatabase;
anti-HLA-DR clone L243, https://www.biolegend.com/de-at/products/purified-anti-human-hla-dr-antibody-792;
anti-CD45 clone D058-1283, https://www.nhpreagents.org/ReactivityDatabase;
anti-NKp44 clone p44-8, https://www.biolegend.com/nl-be/products/purified-anti-human-cd336-nkp44-antibody-3846;
anti-CD163 clone GHI/61, https://www.nhpreagents.org/ReactivityDatabase;
anti-CD73 clone AD2, https://www.nhpreagents.org/ReactivityDatabase;
anti-CD184(CXCR4) clone 12G5, https://www.nhpreagents.org/ReactivityDatabase;
anti-CD141 clone 1A4, https://www.nhpreagents.org/ReactivityDatabase;
anti-CD206 clone 19.2, https://www.nhpreagents.org/ReactivityDatabase;
anti-CD3 clone SP34-2, https://www.nhpreagents.org/ReactivityDatabase;
anti-CD20 clone 2H7, https://www.nhpreagents.org/ReactivityDatabase;
anti-NKG2A clone Z199, https://www.nhpreagents.org/ReactivityDatabase;

anti-CD1a clone O10, https://www.novusbio.com/products/cd1a-antibody-o10_nbp2-34697af488;
anti-CD33 clone AC104.3E3, https://www.nhpreagents.org/ReactivityDatabase;
anti-CD16 clone 3G8, https://www.nhpreagents.org/ReactivityDatabase;
anti-CD11c clone 3.9, https://www.nhpreagents.org/ReactivityDatabase;
anti-CD11b clone ICRF44, https://www.nhpreagents.org/ReactivityDatabase;
anti-CD1c clone L161, https://www.nhpreagents.org/ReactivityDatabase;
anti-TNF clone MAb11, https://www.nhpreagents.org/ReactivityDatabase;
anti-IL-10 clone JES3-9D7, https://www.biolegend.com/fr-fr/products/purified-anti-human-il-10-antibody-1342;
anti-CD107 clone H4A3, https://www.nhpreagents.org/ReactivityDatabase;
anti-IFN-γ clone B27, https://www.nhpreagents.org/ReactivityDatabase;
anti-IL-17 clone BL168, https://www.nhpreagents.org/ReactivityDatabase.

Eukaryotic cell lines

Policy information about cell lines and Sex and Gender in Research

Cell line source(s)

TZM-bl cells were obtained from Dr John C. Kappes, Dr. Xiaoyun Wu and Tranzyme Inc. (courtesy of NIH HIV Reagent Program, currently BEI Resources). The cell line was engineered from the a HeLa cell line (human), which was derived from a female donor.
THP-1 cells were obtained from Millipore Sigma (Burlington, MA, USA).
CEM.NKR.CCR5 cells were obtained from the NIH AIDS Reagent Program, Division of AIDS, NIAD, NIH courtesy of Dr. Alexandra Trkola.
GFP-expressing EGFP-CEM-NKr-CCR5-SNAP cells were kindly provided by Dr. George K. Lewis (PMID: 26969387)

Authentication

Cells lines were not authenticated

Mycoplasma contamination

TZM-bl cell line was tested routinely for mycoplasma and tested negative. The other cell lines were not tested for mycoplasma contamination.

Commonly misidentified lines
(See ICLAC register)

Not Applicable

Animals and other research organisms

Policy information about studies involving animals; ARRIVE guidelines recommended for reporting animal research, and Sex and Gender in Research

Laboratory animals

The research reported in the manuscript complies with all National Institutes of Health ethical regulations and it was approved by the Center for Cancer Research non-human primate animal study protocol prospective scientific committee.
The animals enrolled in the study were Indian rhesus macaques (Macaca mulatta). Macaques were provided by Alpha Genesis Inc. (Yemasee, SC) and Primate Products Inc. (Immokalee, FL) and were housed at the National Institutes of Health (Bethesda, MD).

SIV-based immunization study:
Twelve female macaques with average age of 4.17 years (Standard deviation 0.51) were included in the ALFQA group.
Thirty female macaques with average age of 3.24 years (Standard deviation 0.61) were included in the Alum group.
Mucosal samples from the rectum were obtained from twelve female macaques with average age of 3.44 years (Standard deviation 1.35)

HIV-based immunization study:
Twelve macaques with average age of 3.32 years (Standard deviation 0.31) were included in the HIV-based immunization study. Six macaques (three males and three females) were included in the ALFQA group. Six macaques (three males and three females) were included in the Alum group.

Wild animals

The study did not involve wild animals

Reporting on sex

Animals in the SIV-based immunization study were all females. One of the aims of the study was evaluating the vaccine efficacy following vaginal exposure, therefore only female animals were included in the study.

Animals in the HIV-based immunization study were mixed males and females

Field-collected samples

The study did not involve samples collected from the field

Ethics oversight

The research reported in the manuscript complies with all National Institutes of Health ethical regulations and it was approved by the Center for Cancer Research non-human primate animal study protocol prospective scientific committee. The animals enrolled in the study were Indian rhesus macaques (Macaca mulatta). Macaques were provided by Alpha Genesis Inc. (Yemasee, SC) and Primate Products Inc. (Immokalee, FL) and were housed at the National Institutes of Health (Bethesda, MD) and handled in accordance with the standards of the Association for the Assessment and Accreditation of Laboratory Animal Care (AAALAC) in an AAALAC-accredited facility (OLAW, Animal Welfare Assurance A4149-01). Animal care and procedures were performed under animal study protocols approved by the NCI Animal Care and Use Committees (ACUC; Protocol numbers: VB-013, VB026, VB034, VB042 and VB047). Animals were monitored daily for any signs of illness, and appropriate medical care was provided as needed. Animals were socially housed

per the approved ACUC protocol and social compatibility except during the viral challenge phase when they were individually housed. All clinical procedures, including biopsy collection, administration of anesthetics and analgesics, and euthanasia, were conducted under the direction of a laboratory animal veterinarian.

Note that full information on the approval of the study protocol must also be provided in the manuscript.

Plants

Seed stocks: Not Applicable

Novel plant genotypes: Not Applicable

Authentication: Not Applicable

Flow Cytometry

Plots

Confirm that:

☒ The axis labels state the marker and fluorochrome used (e.g. CD4-FITC).

☒ The axis scales are clearly visible. Include numbers along axes only for bottom left plot of group (a 'group' is an analysis of identical markers).

☒ All plots are contour plots with outliers or pseudocolor plots.

☒ A numerical value for number of cells or percentage (with statistics) is provided.

Methodology

Sample preparation: Eleven freshly collected rectal biopsies were digested with collagenase (2 mg/ml; Sigma-Aldrich) in RPMI (Gibco) without FBS for 1 h at 37ºC. Following incubation, pinches were mechanically separated by using a 10ml syringe with a blunt head canula, cells were washed with R10 and passed through 70µm cell strainer. Cells were counted and used for the experiment. For each sample, 10-15 million cells were recovered from the pinches.

Phenotyping of innate lymphoid, myeloid and dendritic cells in rectal mucosa.
Two million cells were used for phenotype analysis. Cells were stained with Live/Dead blue dye (cat. #L34962, 0.5 µl) from Thermo Fisher, followed by surface staining for 30 minutes at RT with the following antibodies: BB700 anti-CD14 (M5E2; cat. # 745790, 5µl), APC anti-CCR2 (48607; cat. # 558406, 5µl), APC-Cy7 anti-HLA-DR (L243; cat. # 335796, 5µl), BV480 anti-CD45 (D058-1283; cat. # 566145, 5µl), BV650 anti-NKp44 (p44-8; cat. # 744302, 5µl), BV750 anti-CD163 (GHI/61; cat. # 747185, 5µl), BUV493 anti-CD73 (AD2; cat. # 750061, 5µl), BUV563 anti-CD184(CXCR4) (12G5; cat. # 741400, 5µl), BUV661 anti-CD141 (1A4; cat. # 741650, 5µl), BUV737 anti-CD206 (19.2; cat. # 741860, 5µl), BUV805 anti-CD3 (SP34-2; cat. # 742053, 5µl), BUV805 anti-CD20 (2H7; cat. # 612905, 5µl) from BD Biosciences (San Jose, California, USA); PE-Cy7 anti-NKG2A (Z199; cat. no. B10246, 5 µl) from Beckman Coulter (Brea, California, USA); AF488 anti-CD1a (O10; cat. No. NBP2-34697AF488, 5 µl) from Novus Biologicals (Centennial, Colorado, USA); PE anti-CD33 (AC104.3E3; cat. #130-113-349, 5µl) from Miltenyi Biotec (Bergisch Gladbach, North Rhine-Westphalia, Germany); and PE/Dazzle594 anti-CD16 (3G8; cat. # 302054, 5µl), PE-Cy5 anti-CD11c (3.9; cat. # 301610, 5µl), BV570 anti-CD11b (ICRF44; cat. # 301325, 5µl), BV605 anti-CD1c (L161; cat. # 331538, 5µl) from BioLegend (San Diego, California, USA).

Cytokine expression upon stimulation of innate lymphoid cells in rectal mucosa.
Two million rectal mucosal cells were stimulated for 2 h at 37ºC with overlapping gp120 peptides encompassing the sequence of SIVmac251 gp120 or HIV-1 A244 gp120 (2 µg/ml), or 1X PMA/Ionomycin (eBioscience cell stimulation cocktail, Cat. # 00-4970-93 Invitrogen). Subsequently, GolgiPlug protein transport inhibitor (containing Brefeldin A) (cat. #555029, 1 µl) and GolgiStop protein transport inhibitor (containing Monensin) (cat. #554724, 0.7 µl) were added and culturing continued for 18 hours. Following incubation, cells were stained with Live/Dead blue dye (cat. #L34962, 0.5 µl) from Thermo Fisher, followed by surface staining for 30 minutes at room temperature. Surface staining was conducted as described above for phenotyping of innate lymphoid, myeloid and dendritic cells in rectal mucosa. Following surface staining, cells were fixed and permeabilized with a FOX3-transcription buffer set (cat. #00-5523-00) from eBioscience (San Diego, California, USA) according to the manufacturer's recommendation and subsequently intracellular staining with the following: R718 anti-TNF? (MAb11; cat. #566957, 5µl), BV711 anti-IL-10 (JES3-9D7; cat. # 564050, 5µl), BV786 anti-CD107 (H4A3; cat. # 563869, 5µl), BUV395 anti-IFN-γ (B27; cat. # 563563, 5µl) from BD Biosciences (San Jose, California, USA); and BV421 anti-IL-17 (BL168; cat. # 512312, 5µl) from BioLegend (San Diego, California, USA).

Instrument: Samples were acquired on a BD FACSymphony A5 cytometer.

Software: Sample were analyzed with FlowJo software 10.6.

Cell population abundance

Cells for flow cytometry assays were not purified.
For mucosal cells isolation, eleven freshly collected rectal biopsies were digested with collagenase (2 mg/ml; Sigma-Aldrich) in RPMI (Gibco) without FBS for 1 h at 37ºC. Following incubation, pinches were mechanically separated by using a 10ml syringe with a blunt head canula, cells were washed with R10 and passed through 70µm cell strainer. Cells were counted and used for the experiment. For each sample, 10-15 million cells were recovered from the pinches.

Gating strategy

NKG2A+ NK cells were gated as singlets/live/CD45+/CD3-CD20-/CD11b-/NKG2A+NKp44- cells. NKp44+ cells were gated as singlets/live/CD45+/CD3-CD20-/CD11b-/NKG2A-NKp44+ cells. NKG2A- NKp44- cells were gated as singlets/live/CD45+/CD3-/CD20-/CD11b-/NKG2A-NKp44- cells. CD73+ macrophages were gated as singlets/live/CD45+/CD3-CD20-/CD11b+/HLA-DR+/FSC-AHighSSC-AHigh/CD163+/CD73+ cells and expressed as frequency of parental population. Dendritic DC-10 cells were gated as previously described [28] as singlets/SSChighFSChigh/live/CD45+/CD3-CD20-/HLA-DR+/ CD1c-/CD11b+/CD11c+/CD14+CD16+/CD163+/CD141+/CD1a- and expressed as frequency of CD45+ cells.
Since the protocol allowed to collect a maximum number of 11 biopsies of rectal mucosa, which usually yield a cell number ranging between 10 to 15 million, it was not possible to perform concomitant gate validation. Therefore, validation of the gates for NKG2A, Nkp44, CD163, CD141 and CD73 was performed at a later time point and using mucosal samples collected from two animals enrolled in another study. Briefly, following cell isolation, cells were divided into 6 tubes. One tube was fully stained following the procedure described above, whereas the other 5 were stained with all the antibodies minus one constituting the fluorescence minus one (FMO) tubes of NKG2A, Nkp44, CD163, CD141 and CD73 antibodies. Examples of the gating in full stained and FMO tubes are reported in Supplementary figure 3b. Further validation of the gates position in the original dataset was identified by gating each marker on live CD45+ cells. Briefly, using the same FCS files used to generate the data reported in here, each marker was gated in the total live CD45+ population (Supplementary figure 3c). The presence of a high number of positive and negative cells in the CD45+ population allowed an accurate positioning of the gates. The generated gates were then applied in the full gating strategies (Supplementary figure 3a). Examples of the gating on live CD45+ cells are reported in Supplementary figure 3c. Vaccine-induced frequencies of cells were calculated by subtracting the values obtained for samples collected following last immunization of those obtained for samples collected at baseline.

Cytokines were gated on parent population in NKG2A+ NK cells, NKp44+ and NKG2A- NKp44- cells.

☒ Tick this box to confirm that a figure exemplifying the gating strategy is provided in the Supplementary Information.
